# Structure of orbitofrontal cortex predicts social influence

**DOI:** 10.1016/j.cub.2012.01.012

**Published:** 2012-02-21

**Authors:** Daniel K. Campbell-Meiklejohn, Ryota Kanai, Bahador Bahrami, Dominik R. Bach, Raymond J. Dolan, Andreas Roepstorff, Chris D. Frith

**Affiliations:** 1 Center for Neural Science, New York University, 4 Washington Place, NY 10003, USA; 2 Danish Neuroscience Centre, Aarhus University, Nørrebrogade 44, 10 G, Århus Universitetshospital, Århus Sygehus, Aarhus, Denmark; 3 UCL Institute of Cognitive Neuroscience, University College London, 17 Queen Square, London WC1 N 3AR, UK; 4 Wellcome Trust Centre for Neuroimaging, University College London, 12 Queen Square, London WC1 N 3BG, UK

## Abstract

Some people conform more than others. Across different contexts, this tendency is a fairly stable trait [1]. This stability suggests that the tendency to conform might have an anatomical correlate [2]. Values that one associates with available options, from foods to political candidates, help to guide choices and behaviour. These values can often be updated by the expressed preferences of other people as much as by independent experience. In this correspondence, we report a linear relationship between grey matter volume (GM) in a region of lateral orbitofrontal cortex (lOFC_GM_) and the tendency to shift reported desire for objects toward values expressed by other people. This effect was found in precisely the same region in each brain hemisphere. lOFC_GM_ also predicted the functional hemodynamic response in the middle frontal gyrus to discovering that someone else's values contrast with one's own. These findings indicate that the tendency to conform one's values to those expressed by other people has an anatomical correlate in the human brain.

## Main Text

In a functional magnetic resonance imaging (fMRI) study [3], we found that the brain's hemodynamic response to other peoples' preferences for music with respect to one's own, and the influence of those preferences on the brain's value-mediated response to receiving that music, correlate with the tendency to modify our values to accord with the desires of others. This highlighted some physiological dynamics of social influence on value in the brain, but did not address the structural foundations that would link social influence to developmental and evolutionary theory. Moreover, we could not reliably investigate the blood oxygenation level dependent (BOLD) signal from orbitofrontal cortex (OFC) because of signal dropout and distortion in the region. Lesion studies suggest that OFC is causally involved in central components of social influence on value. Damage to this region impairs one's ability to correctly assign value to stimuli, respond appropriately to social cues, and act appropriately during social interaction [4–7]. To investigate the relationship of OFC structure to social influence, we used volumetric-based morphometry (VBM) methods, which are unaffected by signal dropout and distortion, in the same 28 healthy adult subjects (five male) of our previous study [3]. This tested for a correlation between GM of the OFC and the tendency to conform to preferences of others (see Supplemental Experimental Procedures in the Supplemental Information available online).

A week prior to testing, subjects provided the names of twenty pieces of music that they would like to own, but did not own yet. On the test day, subjects rated each submitted song for desirability, from 1 (low) to 10 (high). Next, subjects were told that two music ‘reviewers’, of whom subjects had read descriptions and rated as capable of choosing enjoyable music, had listened to each song; the subjects then performed the task illustrated in Supplemental Figure S1. During a trial, subjects indicated their preference, given a choice of a song they had submitted and an alternative song, which they had never heard. Subjects were then told which song the reviewers preferred. Each submitted song was evaluated relative to six alternatives. After the task, subjects re-rated their desire for each submitted song. Change in desire was tested for a linear relationship with net reviewer preference for the song (times preferred – times not preferred). The resulting coefficient, B_inf_ (M 0.091, SD 0.17), provided a measure of tendency to conform values to values expressed by the reviewers for each subject [3].

**Figure 1:**
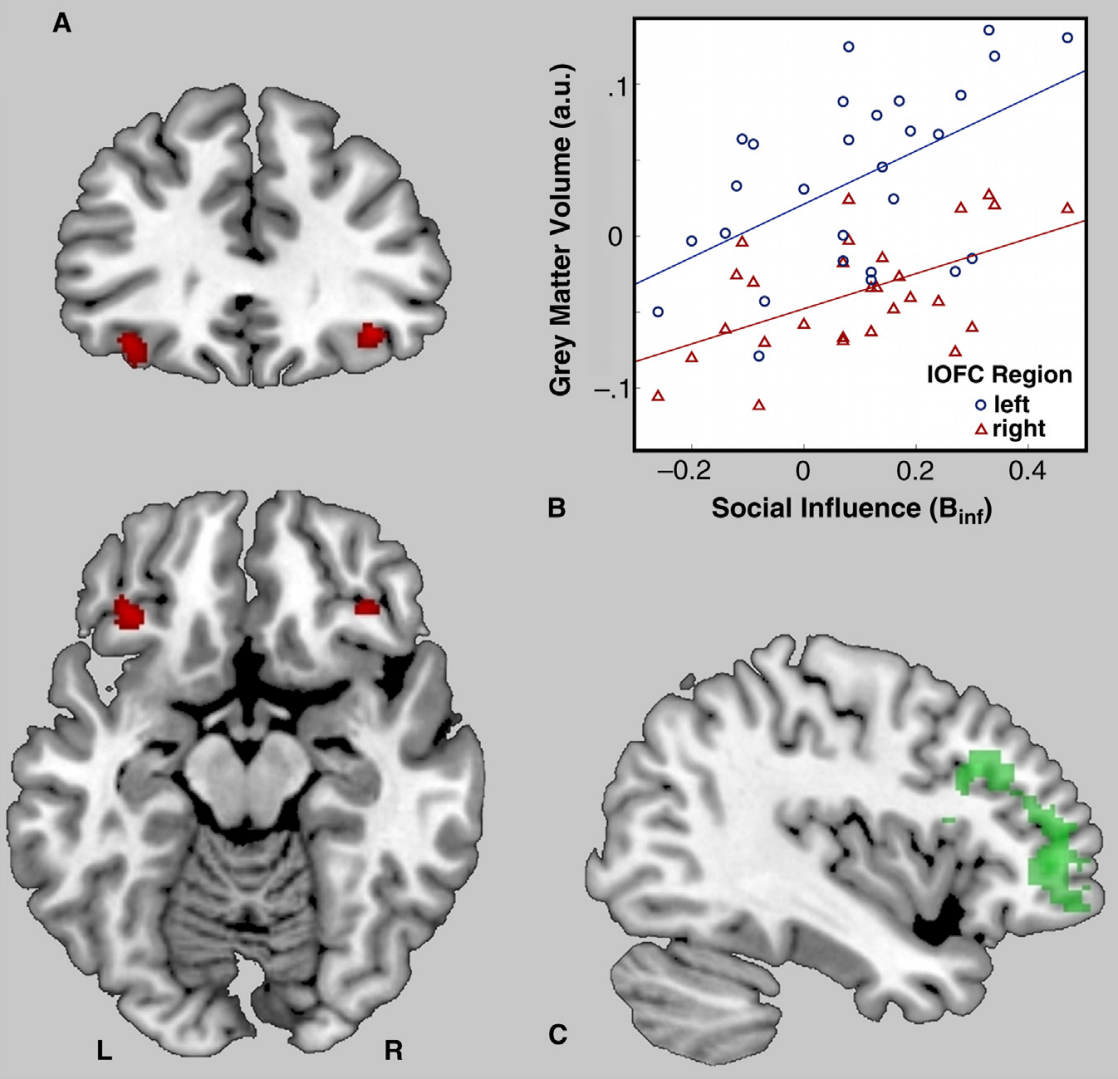
Structural and overlapping functional correlates of social influence. Statistical maps are overlaid onto a standard MNI brain at coordinates: –40 mm, 33 mm, –4 mm. (A) OFC regions in which GM correlated with social influence on value (B_inf_) (threshold p < 0.001, FWE corrected at p < 0.05; right peak 36 mm 33 mm –10 mm, *P*_FWE_ = 0.023, T = 5.56, 73 voxels; left peak –33 mm 28 mm –16 mm, *P*_FWE_ = 0.029, T = 5.43, 183 voxels. (B) Mean GM value (a.u.) within the entire right cluster (red triangles) and entire left cluster (blue circles) plotted against social influence on value (B_inf_). GM values were mean corrected. (C) Overlap (green) in middle frontal gyrus functional activity predicted by conjunction of lOFC_GM_ and B_inf_ during disagreement with experts (vs. agreement) about object value (peak: –40 mm 46 mm 4 mm, Z = 3.72, 768 voxels).

Using VBM, we tested the relationship between B_inf_ and OFC GM. Age, gender, total brain GM and B_inf_ were entered into a multiple regression to OFC GM using T1-weighted MRI images. B_inf_ was linearly related to GM in a specific lateral OFC region (lOFC_GM_) in both hemispheres (Figure 1A,B). No other regions correlated with B_inf_ in a separate whole-brain search, even at a reduced statistical threshold. A functional analysis (see Supplemental Experimental Procedures and Data) found that like B_inf_ [3], lOFC_GM_ predicted the functional hemodynamic response to disagreement with the reviewers about song value, in the middle frontal gyrus (Figure 1C). This is novel evidence of an anatomical correlate of social influence on value. lOFC_GM_ correlated with the tendency to modify desire for objects to accord with values expressed by others.

Intriguingly, the anatomical correlate of social influence was found in lateral rather than medial OFC, regions that are historically associated with monitoring of value [6]. This may be a clue as to the precise role of lateral OFC in social influence. Prior research suggests that lateral OFC is particularly tuned to punishment — when values require updating [6,7]. In this study, lOFC_GM_ predicted the brain's response to disagreement about value. Correspondingly, lOFC_GM_ may index the salience of information that conflicts with one's own (in this case, the opinions of others) or interpretation of this conflict as a punishing event. It could also index the ability to credit detected changes of value to the appropriate option, in line with competing views of lOFC function [7]. Either case might also apply in non-social contexts.

lOFC_GM_ could reflect a single psychological trait that directly corresponds to behavior ranging from conformity to anticonformity (for example, affinity for group membership). On the other hand, it is possible that conformity and anticonformity are mediated by two separate mechanisms. For example, lOFC_GM_ could mediate only conformity-related cognition that, when reduced, allows anticonformity-related cognition to have a greater impact on behavior (negative B_inf_ coefficients). It seems unlikely that conformity and anti-conformity are completely independent given that functional [3] and anatomical correlates span the whole range of positive and negative B_inf_ values in a single brain region. However, this possibility is consistent with results obtained in a subsample of 23 subjects with B_inf_ scores near zero or above, and in all 28 subjects when anticonforming changes of value are coded as independence (no change) (see Supplemental Information). The precise conformity-related cognition associated with lOFC_GM_ is now an enticing question for future research.

Clinically, apparent changes of social conduct that result from atrophy or damage to prefrontal cortex [4,6] might result from a reduced tendency to adopt or even respond to values expressed by others. Developmentally, increases of conformity during preadolescence and decreases during adolescence [8] may relate directly to tandem changes in prefrontal cortex grey matter volume [9]. In evolution, the findings are consistent with the view that expansion of primate cortex may relate to a greater capacity for social learning [10]. Finally, an anatomical correlate of social influence on value suggests the existence of a biological basis for individual differences of a basic ability that allows us to represent, update and integrate the values of others in order to learn socially and manage one's reputation.

The editors of *Current Biology* welcome correspondence on any article in the journal, but reserve the right to reduce the length of any letter to be published. All Correspondence containing data or scientific argument will be refereed. Queries about articles for consideration in this format should be sent by e-mail to cbiol@current-biology.com

## Supplemental Information

Document S1. Figure S1 and S2, Supplemental Data, Supplemental Experimental Procedures, and Supplemental References
