# Supplementary material for: Structure of orbitofrontal cortex predicts social influence
Source: Curr Biol. 2012 Feb 21;22(4):R123–4. doi: 10.1016/j.cub.2012.01.012 (PMC3315000; doi:10.1016/j.cub.2012.01.012)
Supplement: Document S1. Figure S1 and S2, Supplemental Data, Supplemental Experimental Procedures, and Supplemental References [file mmc1.pdf]

Supplemental Information: Structure of Orbitofrontal Cortex Predicts Social Influence

Daniel K Campbell-Meiklejohn, Ryota Kanai, Bahador Bahrami, Dominik R Bach, Raymond J Dolan,

Andreas Roepstorff & Chris D. Frith

Supplemental Experimental Procedures

Experimental procedures have been described previously [S1] and are reproduced here. The only differences are the Volumetric Morphometry Based methods (VBM) and conjunction functional magnetic resonance imaging (fMRI) analysis described at the end of this section.

Subjects

Twenty-eight neurologically and psychologically healthy (15 males; mean age 26. ± 5.24 (S.D.); age range 19 to 39, all right-handed) gave informed consent and participated in the study. Each was recruited via public advertisements in the London, UK. Subjects had normal or corrected-to-normal vision. The UCL Research Ethics Committee approved this study. Subjects received 20 British pounds sterling in guaranteed payment and 10 songs on a CD.

Experimental Procedures

One week prior to scanning, subjects submitted a list of twenty songs that could be purchased from an online music store. Each was a song that the subject desired but did not yet own. On arrival to the centre, subjects had their photo taken and rated each of their 20 songs for desirability on a scale from 1 (I do not want this song) to 10 (I really want this song). Subjects also looked at pictures of two music 'experts' and read descriptions of the two them, as follows:

> Dave is a respected musician and sometimes London DJ. He has been listening to and playing music as long as he can remember. He owns a massive collection of music from over 50 countries, but he also listens to the top 40 at work. He is an avid drummer and plays guitar. When DJing, he creatively mixes samples from anything from hip hop to the Beatles and describes his music taste as "eclectic but with a good ear for quality sounds."
>
> Michelle is a music writer. Michelle is always listening to music. She reviews albums for UK and USA music magazines, interviews up-and-coming artists and often has access to music well before the general public. She describes her music taste as very open, and listens to a wide

variety. She likes new and independent artists, but admits that she also listens pop music while out and about in town and with friends.

Subjects were asked to rate each reviewer from 1 (not at all) to 7 (very much) for how much the person could be trusted to pick music that the subject would like. No comparisons were made between experts during the study. Descriptions were created to communicate a degree of expertise across a broad range of popular music tastes.

Subjects were informed that the two experts had listened to the 20 songs and provided reviews for each. Reviews were preferences between each of the 20 subject-provided songs and an alternative song, provided by the experimenter. Each subject-provided song was reviewed six times (relative to six different alternative songs). Subjects received instructions for the task and answered a series of questions to confirm that their task was understood. Each subject confirmed that they believed the reviews were real.

On arrival to the center, subjects had their photo taken and rated each of their 20 songs for desirability on a scale from 1 (I do not want this song) to 10 (I really want this song). They then read descriptions and viewed pictures of the two expert music reviewers before rating each reviewer from 1 (not at all) to 7 (very much) for how much they would trust that reviewer to pick a song that they (the subject) would like. The mean reviewer rating was 4.43 ± .91 on a scale from 1 ('very unlikely') to 7 ('very likely') that the reviewer would choose a song that the subject would like). The mean male reviewer's rating was 4.61 ± 1.1 and the mean female reviewer's rating was 4.27 ± 1.1. Thus subjects perceived both reviewers as capable of choosing music that the subject would like.

Subjects then performed the task. After completing the task, subjects rated each of their 20 songs for desirability for a second time. The mean song desirability rating was 7.4 ± .07 before the experiment and 7.61 ± 1.6 after the experiment. Subjects were also asked if they had learned more about the reviewers or more about the songs. The 10 songs for which the subject had the most tokens (from the object outcome of the task) were purchased for the subject.

Influence Task

The task was programmed and run using Presentation v.12 (Neurobehavioural Systems). Visual displays were back-projected to a display in the scanner. Subjects viewed the displays via a mirror placed above their eyes. Responses (from the right hand) were collected using by two fibre-optic button boxes.

Each trial (see Figure S1) began with a choice for the subject. We presented subjects with two songs at the top of the screen. One was a song that the subject provided. The other was an alternative, provided by the experimenter. The alternative was a Canadian or Scandanavian pop song, which was real but unknown to the subject (confirmed after the scan session). Song choices were randomly assigned to the left and right side of the display. Pictures of the experts were arranged vertically down the center of the display. A picture of the subject appeared at bottom of the screen, beneath the expert pictures. The words "I prefer" were placed under each photo. The subject's task was to move their own picture beneath the song they desired the most. Subjects pressed the left button to move their picture left, or the right button to move it right. A scrambled picture of the subject was placed under the song they did not choose. Subject-provided songs appeared equally-often on left and right sides of the display.

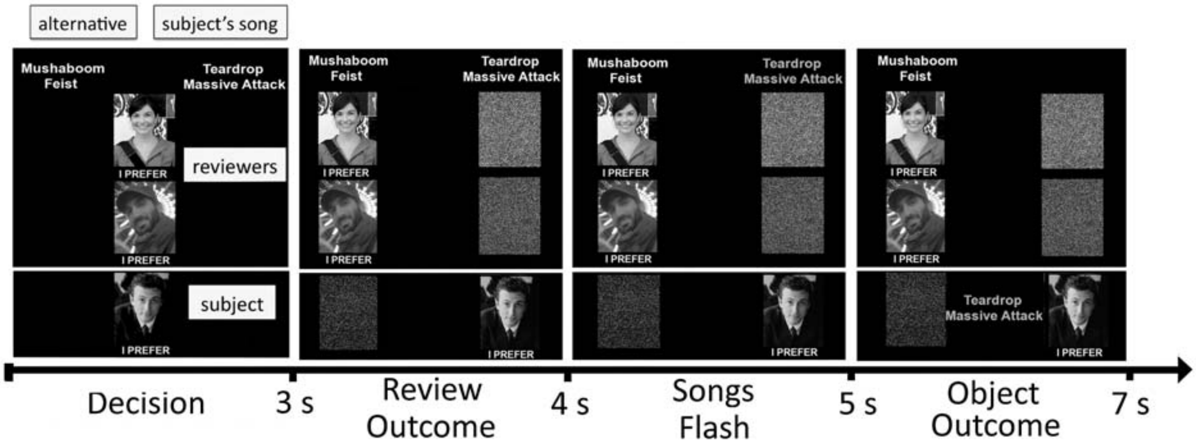

Figure S1. Task Display Sequence: The subject indicates own preference by moving own picture under choice (decision), is informed of the preference of the experts (review) and receives a token for one of the songs (object outcome). (s) = time in seconds. 120 trials.

Subjects were told that the song that they chose had a slightly (less than 5%) higher chance of being chosen for a token at the end of the trial to provide motivation to pick their real preference. Each song actually had a 50% chance of being chosen. Subjects knew that the songs with the most tokens at the end of the task were to be purchased for them and placed on a CD. There was a time limit of 2 seconds to make a choice. If no choice was made, a large 'X' appeared on the screen for the remainder of the trial.

After making their choice, subjects learned about the expert's opinions. The pictures of each expert were moved under their respective preference. Scrambled pictures of the experts were placed under songs they did not choose. Experts could both prefer the subject-provided song, both prefer the alternative, or disagree with each other. This phase is termed the 'review outcome.' Next, the songs alternately changed color between green and white (every 50ms, for 1s). Finally, a song was chosen for a token and appeared at the bottom of the screen. This phase was the 'object outcome.'

Expert reviews were completely independent from object outcomes. During instruction, subjects confirmed that expert choices did not predict which song token would be received. The subject received a token for each of their submitted songs as often as they received an alternative. The order of trials was optimized to provide maximum efficiency for detection of Blood Oxygenation Level Dependent (BOLD) activity related, independently, to different review and object outcomes. For these purposes, it was not possible to use real expert reviews, and confederate reviews were used in their place. Each participant confirmed that they believed the reviews were real and were debriefed at the end of the experiment. As a result, trials could be placed close together in time with a brief minimum of 3 seconds between each modeled event (see section on fMRI analysis) reducing subject time in the scanner but still controlling for nonlinearities of the BOLD signal.

Decisions appeared at time 0 of each trial. Review outcomes appeared at 3 seconds, and songs began to flash at 4 seconds. Object outcomes were presented at 5 seconds and remained on display for 2 seconds. A fixation cross was displayed for 2 seconds between each trial.

There is no non-social equivalent, to our knowledge, to a human opinion. Even a 'computer' from which one could make accurate inferences of subjective human value only acts as an indirect inference of the human opinions used to program it, and thus computer reviews would still remain 'social.' For this reason, we saw little merit in providing an artificial 'non-social' control in this study.

Post Scanning

After completing the task, subjects rated each of their 20 songs for desirability for a second time. Subjects were also asked if they had learned more about the reviewers or more about the songs. The

10 songs for which the subject had the most tokens (from the object outcome of the task) were purchased for the subject.

Social Influence Measure

The measure of trait social influence, B_{inf} was calculated for each subject as the Pearson's product-moment correlation coefficient (Pearson's *r*) for the bivariate correlation between change in song desirability (post task rating – pre task rating) and net preference of reviewers (times preferred - times not preferred), calculated in SPSS (SPSS 19.0). This value can also be interpreted as the expected change, in standard deviations, of subject desire for a song for every standard deviation of net preference of reviewers (the standardized Beta coefficient of a linear regression between the two variables).

MRI Data Acquisition

Scanning took place at the Wellcome Trust Centre for Neuroimaging in London, UK. Subjects were scanned at 3 Teslas with a Siemens MAGNETOM Trio scanner (Siemens Medical Solutions, Erlangen, Germany) fitted with a 12-channel head coil. 176-slice whole-brain anatomical scans (matrix, 256*256; 1mm slice thickness; TE, 2.48ms; TR=7.92ms; flip angle, 16°; TI=910ms) were acquired using a modified driven equilibrium Fourier transform (MDEFT) sequence with optimized parameters as described previously [S2]. Functional data was collected as T2-weighted echo planar images (EPI) in descending slice acquisition order. Each volume (voxel size: 3*3*3mm; TE, 30 ms; TR, 3360ms) contained 48 slices, covering the whole brain. We attempted to minimize BOLD sensitivity losses in the orbitofrontal cortex due to susceptibility artefacts were minimized by applying a z-shim gradient moment of -1.4 mT/m*ms, a slice tilt of -30°and a positive PE gradient polarity [S3], but dropout and

distortion was still present. Images were reconstructed by performing a standard 3D Fourier Transform, followed by modulus calculation. No data filtering was applied in k-space or in the image domain. Field maps were acquired with a standard double echo gradient echo field map sequence (TE, 10.0 and 12.46 ms), using 64 slices covering the whole head (voxel size, 3*3*2 mm with 1mm gap between slices).

VBM Analysis

These structural images were analysed by voxel-based morphometry [S4]. T1-weighted MR images were first segmented for grey matter and white matter using the segmentation tools in SPM8 (http://www.fil.ion.ucl.ac.uk/spm). Subsequently, we performed Diffeomorphic Anatomical Registration Through Exponentiated Lie Algebra (DARTEL) [S5] in SPM8 for inter- subject registration of the GM images. The registered images were smoothed with a Gaussian kernel (FWHM = 8 mm) and were then transformed to MNI stereotactic space using affine and non-linear spatial normalisation implemented in SPM8. To ensure that the local grey matter volume was retained before and after spatial transformation, the image intensity was modulated by the Jacobian determinants of the deformation fields. Thus the value of grey matter volume (GM) represented the tissue volume per unit of spatially normalised image in arbitrary units. These final images were entered into a multiple regression analysis across subjects. Regressors of gender, age and the total brain GM were included in the design matrix as covariates of no interest and thus regressed out any effects correlated with these factors. Finally a B_{inf} was entered as a regressor of interest. Resulting voxels positively related to B_{inf} were thresholded at $p < 0 .001$ with a minimum cluster size of 20 voxels. With our specific anatomical hypothesis, voxels surpassing this stringent threshold were corrected for multiple comparisons with a strict family-wise error (FWE) correction at a $P < 0.05$ threshold for significance within a mask of the

orbitofrontal cortex derived from the Oxford-Harvard Cortical Structural Atlas (www.fmrib.ox.ac.uk/fsl).

fMRI Analysis

All subject-level standard pre-processing, modelling and contrasts for the fMRI analysis are exactly as described previously [S1].

Preprocessing

Image unwarping, and motion correction was performed using statistical parametric mapping (SPM 5; Wellcome Trust Centre for Neuroimaging; www.fil.ion.ucl.ac.uk/spm) on Matlab (version 7.1, MathWorks). EPI images were generated off-line from the complex k-space raw data using a generalized reconstruction method based on the measured EPI k-space trajectory to minimize ghosting. They were then corrected for geometric distortions caused by susceptibility-induced field inhomogeneities. A combined approach was used which corrects for both static distortions and changes in these distortions due to head motion [S6, S7]. The static distortions were calculated for each subject from a field map that was processed using the FieldMap toolbox as implemented in SPM5. Using these parameters, the EPI images were then realigned and unwarped with a procedure that allows the measured static distortions to be included in the estimation of distortion changes associated with head motion. The remaining preprocessing and was carried out with the FMRIB's Software Library (FSL) version 5.63 [S8]. Brain matter was segmented from non-brain using a mesh deformation approach [S9]. High pass temporal filtering was applied using a Gaussian-weighted running lines filter, with a cut-off of 50s [S10]. Each volume was smoothed with a Gaussian filter (full-width half-maximum of 5mm). Independent Component Analysis was used to visually identify and

remove artefacts in the data using Multivariate Exploratory Linear Optimized Decomposition into Independent Components (MELODIC) software [S11].

Single Subject General Linear Models (GLMs)

Modelling and statistical analysis of fMRI data was carried out with the FEAT (FMRI Expert Analysis Tool, www.fmrib.ox.ac.uk/fsl) version 5.63 [S8]. A standard GLM was used for individual subject analyses. The GLM was fit in pre-whitened data space (to account for autocorrelation in the FMRI residuals [S12]). Outcome conditions were: experts prefer the same song as the subject, experts prefer the alternative song, subject receives their preferred song and subject receives the alternative song. These regressors (plus their temporal derivatives) were included in the model as stick functions placed midway through the 'object outcome' display period. Decisions, trials in which subjects took longer than 2s to respond, and trials in which the subject chose the alternative song (i.e. not their pre-submitted song) were included in the model as separate regressors but not used in further analysis. Regressors were convolved with the FSL default haemodynamic response function (HRF, gamma function, delay = 6s, standard deviation = 3 s). High-pass temporal filtering (50s) was also applied to the regressors. GLM results were estimated [S13] and transformed, after spatial normalization, into standard (MNI152) space [S10].

Two subject-level contrasts were used for this study. These contrasts were previously shown to generate BOLD responses that correlate between subjects with B_{inf} [S1]:

1. *Review Outcome:* This contrast highlights BOLD signal differences between outcomes when both experts agreed with the subject preference and outcomes in which both experts preferred the alternative song (conflict of opinion).

2. *Review Outcome x Object outcome (effect reviews on reward activity):* This is the interaction effect between review outcome and object outcome (receiving the preferred song relative to receiving the alternative song). In other words, this contrast highlighted if the BOLD signal difference when receiving the preferred song relative to receiving the alternative song is larger when the experts preferred the same song as the subject (and smaller or inversed when the experts preferred the alternative).

Group Level GLMs

We carried out two group-level analyses with FLAME 1+2 (FMRIB's Local Analysis of Mixed Effects [S8]. For each, we modelled all subjects as a single group and a GLM was fit to the subject-level effects for the contrasts described above. Two regressors were entered into each group-level model – one mean effect and one demeaned parametric regressor. The first model included:

1. Mean effect.
2. Mean $lOFC_{GM}$ value (a.u.) within area of VBM cluster shown to correlate with B_{inf}

The second model included:

1. Mean effect.
2. B_{inf}

Statistical maps were thresholded at a Z score of 2.3 and whole brain cluster-corrected with a cluster significance level of $p < 0.05$ [S14-S16]. We then performed a conjunction analysis of the group level effects of $lOFC_{GM}$ and B_{inf} to test for commonalities based on the conjunction null hypothesis that one or more of the effects are null [S17], with the same threshold and correction.

We did not find a significant interaction between $lOFC_{GM}$ and social influence on the reward response in any area of the brain, including the ventral striatum. To double-check, we performed a simple bivariate correlation analysis across subjects between (i) $lOFC_{GM}$ at the peak voxel associated with B_{inf} and (ii) parameter estimates of the review outcome x object outcome contrast at the peak voxel shown to interact with B_{inf} in the ventral striatum [S1].

Supplemental Data

Anatomical Link to Functional Correlates

We found a significant association between $lOFC_{GM}$ and the functional response to disagreement with others about object value (the review outcome), in the left middle frontal gyrus (peak: -42mm 46 mm 4 mm, Z = 3.92, 1050 voxels). The conjunction analysis revealed considerable overlap between the effects of B_{inf} and $lOFC_{GM}$ on review outcome in the middle frontal gyrus (Peak: -40mm 46mm 4mm, Z = 3.72, 768 voxels). The relationship between $lOFC_{GM}$ and middle frontal gyrus reflects reciprocal interconnections between the regions [S18].

Unlike $B_{inf„}$ $lOFC_{GM}$ did not predict the interaction effect of review outcome x object outcome (i.e. social influence on reward responses) in the ventral striatum, in a whole brain search. Since the middle frontal gyrus review outcome activity also failed to predict social influence on reward response [S1], this might be expected. We interpret activity and structure relating to review outcome as relating to a limiting factor influence that leaves room for yet other factors to affect the impact of the social feedback on reward responses in the striatum. Importantly, we do not conclude that $lOFC_{GM}$ and social influence on striatum reward responses are completely unrelated. Indeed, a correlation between peak voxels of the two effects across subjects approached significance (r = .333, p = 0.083) and encourages further investigation of the relationship.

Conformity, Anticonformity and Independence

Most social influence experiments pit conformity against either anticonformity (change away from the group) or independence (no influence *or* no change) but the difference between these dimensions is often overlooked. Many models of these dimensions have been put forward [S19-S22], with some experimental support for a 'conformity to anticonformity' dimension [S22, S23]. Still, one might argue that changes of value toward and away from values expressed by others are independently mediated by different traits, and therefore different anatomy. If so, one might ask if the results hold when limited to a dimension of conformity-to-independence.

Therefore, in separate analyses, we exclusively analysed 23 subjects with B_{inf} scores near or above zero. We removed 5 subjects with scores more than .1 standard deviation below zero. Anatomical correlations with B_{inf} (right peak: 36 32 -11, 15 voxels, T(18)=4,81, $P_{FWE} < .004$; left peak: -33 30 -11, 30 voxels, T(18) = 4.59, $P_{FWE} < .004$) in this subset were comparable to those resulting from all 28 subjects despite the loss in statistical power (Figure S2A). Using mean grey matter density of these 23 subjects within the regions of $lOFC_{GM}$ clusters as a between-subject parametric regressor in the fMRI contrast of disagreement vs. agreement of these 23 subjects also replicated the findings in all 28 subjects (peak: -38mm 52mm 14mm, 316 voxels, Z = 3.65, P < .05 cluster-corrected).

With another approach, we included all subjects, but set all changes away from others preferences within each subject to 0 (no change), thus limiting value changes to a conformity-independence dimension and maintaining statistical power of the group analysis. This analysis makes the reasonable assumption that, given no option to distance ones responses from preferences of others,

anticonformity would be expressed as independence (i.e. no change). Again, the result remains similar to our original findings (right peak: 39 38 -9, 44 voxels, T (23)= 5.15, $P_{FWE} < .001$; left peak: -33 30 -15, 81 voxels, T(23) = 4.33, $P_{FWE} < .005$) (Figure S2B). Therefore, even if the baseline is independence, lOFC grey matter predicts the degree of social influence toward the opinions of others. Like all studies that use such a baseline, one should not assume that independence means that opinions of others are not attended. We also note that the original analysis (anticonformity-conformity) provides stronger statistical effects and corresponding cluster size.

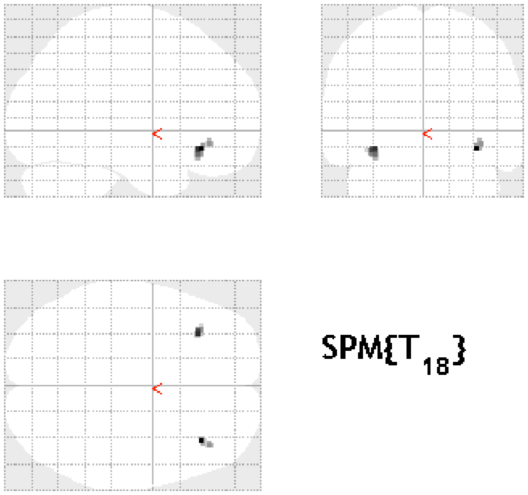

A.

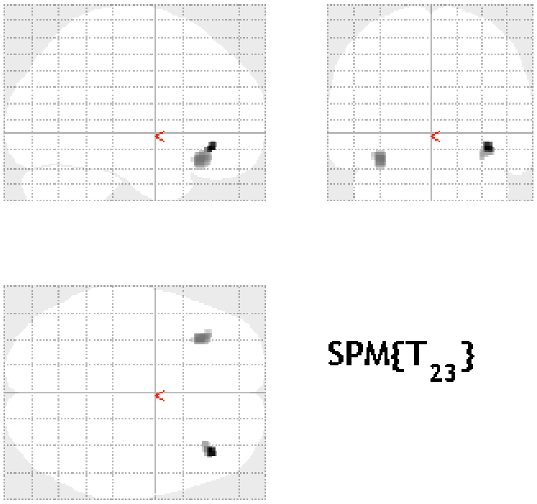

B.

Figure S2: Glass brain statistical parametric maps. A. OFC regions in which GMV correlated with social influence on value (B_{inf}) in 23 subjects with near or above zero B_{inf} scores. **B.** OFC regions in which GMV correlated with social influence on value, where changes of opinion away from the expressed values of others were scored as zero change (independence) using all subjects.

Supplemental References

S1. Campbell-Meiklejohn, D.K., Bach, D.R., Roepstorff, A., Dolan, R.J., and Frith, C.D. (2010). How the opinion of others affects our valuation of objects. Curr. Biol. *20*, 1165-1170.
S2. Deichmann, R., Schwarzbauer, C., and Turner, R. (2004). Optimisation of the 3D MDEFT sequence for anatomical brain imaging: technical implications at 1.5 and 3 T. Neuroimage *21*, 757-767.
S3. Weiskopf, N., Hutton, C., Josephs, O., and Deichmann, R. (2006). Optimal EPI parameters for reduction of susceptibility-induced BOLD sensitivity losses: a whole-brain analysis at 3 T and 1.5 T. Neuroimage *33*, 493-504.
S4. Ashburner, J., and Friston, K.J. (2000). Voxel-based morphometry--the methods. Neuroimage *11*, 805-821.
S5. Ashburner, J. (2007). A fast diffeomorphic image registration algorithm. Neuroimage *38*, 95-113.
S6. Andersson, J., Hutton, C., Ashburner, J., Turner, R., and Friston, K. (2001). Modeling geometric deformations in EPI time series. Neuroimage *13*, 903-919.
S7. Hutton, C., Bork, A., Josephs, O., Deichmann, R., Ashburner, J., and Turner, R. (2002). Image distortion correction in fMRI: A quantitative evaluation. Neuroimage *16*, 217-240.
S8. Smith, S., Jenkinson, M., Woolrich, M., Beckmann, C., Behrens, T., Johansen-Berg, H., Bannister, P., De Luca, M., Drobnjak, I., Flitney, D., et al. (2004). Advances in functional and structural MR image analysis and implementation as FSL. Neuroimage *23 Suppl 1*, S208-219.
S9. Smith, S. (2002). Fast robust automated brain extraction. Hum. Brain Mapp. *17*, 143-155.
S10. Jenkinson, M., Bannister, P., Brady, M., and Smith, S. (2002). Improved Optimization for the Robust and Accurate Linear Registration and Motion Correction of Brain Images. Neuroimage *17*, 825-841.
S11. Beckmann, C., and Smith, S. (2004). Probabilistic independent component analysis for functional magnetic resonance imaging. IEEE Trans. Med. Imaging *23*, 137-152.
S12. Woolrich, M., Ripley, B., Brady, M., and Smith, S. (2001). Temporal Autocorrelation in Univariate Linear Modeling of FMRI Data. Neuroimage *14*, 1370-1386.
S13. Woolrich, M., Behrens, T., Beckmann, C., Jenkinson, M., and Smith, S. (2004). Multilevel linear modelling for FMRI group analysis using Bayesian inference. Neuroimage *21*, 1732-1747.
S14. Grasby, P., Frith, C., Friston, K., Simpson, J., Fletcher, P., Frackowiak, R., and Dolan, R. (1994). A graded task approach to the functional mapping of brain areas implicated in auditory-verbal memory. Brain *117 (Pt 6)*, 1271-1282.
S15. Forman, S.D., Cohen, J.D., Fitzgerald, M., Eddy, W.F., Mintun, M.A., and Noll, D.C. (1995). Improved assessment of significant activation in functional magnetic resonance imaging (fMRI): Use of a cluster-size threshold. Magn. Reson. Med. *33*, 636-647.
S16. Worsley, K.J., Marrett, S., Neelin, P., Vandal, A.C., J, F., and Ac, E. (1996). A unified statistical approach for determining significant signals in images of cerebral activation. Hum. Brain Mapp. *4*, 58-73.
S17. Nichols, T., Brett, M., Andersson, J., Wager, T., and Poline, J.B. (2005). Valid conjunction inference with the minimum statistic. Neuroimage *25*, 653-660.
S18. Cavada, C., Company, T., Tejedor, J., Cruz-Rizzolo, R.J., and Reinoso-Suarez, F. (2000). The anatomical connections of the macaque monkey orbitofrontal cortex. A review. Cereb. Cortex *10*, 220-242.

S19. Nail, P.R., MacDonald, G., and Levy, D.A. (2000). Proposal of a four-dimensional model of social response. Psychol. Bull. *126*, 454-470.

S20. Nail, P.R. (1993). An Analysis and Restructuring of the Diamond Model of Social Response. Personality and Social Psychology Bulletin *19*, 106-116.

S21. Willis, R.H. (1965). Conformity, independence, and anticonformity. Human Relations.

S22. Stricker, L.J., Messick, S., and Jackson, D.N. (1970). Conformity, Anticonformity, and Independence - Their Dimensionality and Generality. J. Pers. Soc. Psychol. *16*, 494-&.

S23. Chu, L. (1979). The Sensitivity of Chinese and American Children to Social Influences. The Journal of Social Psychology *109*, 175-186.
